# Rhinovirus-16 Induced Release of IP-10 and IL-8 Is Augmented by Th2 Cytokines in a Pediatric Bronchial Epithelial Cell Model

**DOI:** 10.1371/journal.pone.0094010

**Published:** 2014-04-04

**Authors:** Julie A. Cakebread, Hans Michael Haitchi, Yunhe Xu, Stephen T. Holgate, Graham Roberts, Donna E. Davies

**Affiliations:** 1 Academic Unit of Clinical and Experimental Sciences, University of Southampton Faculty of Medicine, University Hospital Southampton, Southampton, United Kingdom; 2 NIHR Respiratory Biomedical Research Unit, University Hospital Southampton, Southampton, United Kingdom; University ofTennessee Health Science Center, United States of America

## Abstract

**Background:**

In response to viral infection, bronchial epithelial cells increase inflammatory cytokine release to activate the immune response and curtail viral replication. In atopic asthma, enhanced expression of Th2 cytokines is observed and we postulated that Th2 cytokines may augment the effects of rhinovirus-induced inflammation.

**Methods:**

Primary bronchial epithelial cell cultures from pediatric subjects were treated with Th2 cytokines for 24 h before infection with RV16. Release of IL-8, IP-10 and GM-CSF was measured by ELISA. Infection was quantified using RTqPCR and TCID_50_. Phosphatidyl inositol 3-kinase (PI3K) and P38 mitogen activated protein kinase (MAPK) inhibitors and dexamethasone were used to investigate differences in signaling pathways.

**Results:**

The presence of Th2 cytokines did not affect RV replication or viral titre, yet there was a synergistic increase in IP-10 release from virally infected cells in the presence of Th2 cytokines. Release of IL-8 and GM-CSF was also augmented. IP-10 release was blocked by a PI3K inhibitor and IL-8 by dexamethasone.

**Conclusion:**

Th2 cytokines increase release of inflammatory cytokines in the presence of rhinovirus infection. This increase is independent of effects of virus replication. Inhibition of the PI3K pathway inhibits IP-10 expression.

## Introduction

Asthma is a complex respiratory disease characterized by variable airflow obstruction, bronchial hyper-responsiveness and airway inflammation. In atopic asthma, the Th2 type cytokines interleukin (IL)-13 and IL-4 [1] are key players in allergic responses, playing a pivotal role in inflammatory and remodelling aspects of asthma pathogenesis [2], [3]. Th2 cytokines can impair immune responses to viral infections. It has been shown that adults with a ‘less atopic phenotype’ have a greater ability to clear human rhinovirus (RV) compared to atopic adults [4], [5].

Although atopic asthma is the more dominant form of asthma during school years and into young adulthood [6]–[8], exacerbation of asthma has been strongly linked to respiratory infection alone, with 44% to 80% of childhood asthma exacerbations being triggered by RV infection [9]. RV is the most common pathogen associated with asthma exacerbations in children [10]. Furthermore, a combination of sensitization, high exposure to one or more allergens, *and* viral detection significantly increases the risk of hospitalization for asthma [11], [12].

The mechanisms behind the association of atopy and virus co-morbidity are as yet, unclear. Some studies have provided evidence that Th2 cytokines and viral pathogen associated molecular patterns (PAMPs) trigger release of the Th2 polarizing cytokine thymic stromal lymphopoietin [13], whilst other studies have shown that the epithelial effects of Th2-associated cytokines, such as IL13 and IL4, are dominant over the effects of the Th1-associated cytokines such as interferon-gamma [14]. Like allergen and RV, IL-13 and IL-4 have been shown to enhance expression of ICAM-1 [14], [15], the cellular receptor for major group RVs. Th2 cytokines have the potential to facilitate entry of major group RV into airway cells of atopic subjects and to favour migration and activation of immune effector cells into the airway. This study uses epithelial monolayer cultures from a non asthmatic pediatric population to investigate whether the presence of Th2 cytokines modulates virus-induced inflammation; we also examine the ability of signaling pathway inhibitors to suppress these responses.

## Methods

### Ethics statement

Ethical approval was given by the Southampton and South West Hampshire Research Ethics Committee (07/Q1704/21). Parents of participants provided written informed consent. Procedures, including consent, was approved by the ethics committee.

### Primary Cell culture

Bronchial brushings were obtained by fibreoptic bronchoscopy from pediatric subjects, not diagnosed with asthma, following current guidelines [16]. Subjects were recruited when attending hospital for clinically indicated bronchoscopy or other planned surgical procedure under a general anaesthetic. The subject group comprised 8 males (mean age 7.3 years; range 2–15 years), and 7 females (mean age 8.4 years; range 1–15 years). Details of patient phenotypes are in table 1. Brushings were processed for primary bronchial epithelial cell (pBEC) culture in Bronchial Epithelial Growth Medium (BEGM) (Lonza, Wokingham, United Kingdom,) as previously described [17]. Pediatric pBECs (ppBECs) were plated onto collagen coated (30 μg/ml PureCol, Inamed Biomaterials, Fremont, USA) 12-well plates (Nunc, Fisher Scientific, Loughborough, United Kingdom), at passage 2. For experiments minimal medium, BEBM, was used.

**Table 1:** Summary of clinical and physiological characteristics of subjects studied.

Subject	Age (yrs)	Gender	Indication/operation
1	1	F	Bronchoscopy - recurrent chest symptoms & intermittent stridor
2	11	M	Bronchoscopy - recurrent severe croup
3	3	M	Bronchoscopy - recurrent croup and stridor
4	5	M	Bronchsocopy- recurrent cough, prematurity, tracheomalacia
5	3	M	Bronchoscopy - tracheomalacia
6	1	F	Bronchoscopy - recurrent chronic cough
7	2	M	Bronchoscopy - tracheomalacia
8	6	M	Adenoidectomy and tonsillectomy, gromit insertion
9	14	M	Surgery for nasal septum deviation
10	10	F	Dental procedure
11	12	F	Dental procedure
12	15	F	Dental procedure
13	7	F	Dental procedure
14	13	F	Dental procedure
15	15	M	Dental procedure

Subjects were recruited when attending hospital for clinically indicated bronchoscopy or other planned surgical procedure under a general anaesthetic. Bronchial brushings were obtained by fibreoptic bronchoscopy from pediatric subjects not diagnosed with asthma following current guidelines [16]. Mean age (years) 7.9 (range 1–15), M = 8 F = 7.

### Virus culture

RV16 (a gift from Professor Sebastian L. Johnston, Imperial College, London) was amplified as previously described [18]. Virus strain was confirmed by qPCR (Primer Design, Southampton, UK) and infectivity determined with HeLa titration assay and 50% tissue culture infective dose (TCID_50_)/ml.: Ultra violet treated virus controls (UV) were obtained by irradiating live virus stocks (1200 mJ/cm2 on ice for 50 min). Inactivation of virus was confirmed by TCID_50_.

### Infection of cells with RV

pBECs were infected with RV16 (Dose: 1×10^6^ TCID_50_ units/10^6^ cells) for 1 hour at room temperature. Cells were washed to remove residual viral particles and the cultures replenished with BEBM. Different multiplicities of infection (MOIs) were not tested due to limitations in sample availability. Based on previous experience we chose a moderate dose of virus for infection. RV16 was chosen since it has minimal cytotoxic effects, as seen in figure S1.

### Cytokine treatments

Cultures were pretreated with 10 ng/ml [19] of IL-13, IL-4 or both, for 24 h prior to infection. Cytokines were replenished with BEBM after washing, post-infection.

Controls of UV irradiated RV16, medium alone and Th2 only controls were included in all experiments.

### Inhibitor treatments

Inhibitors were added to cultures 30 minutes before infection with RV16 and were refreshed after infection. Inhibitors of PI3K (LY294002, Calbiochem, Merck Millipore, UK) and p38 (SB03580, Sigma-Aldrich, Dorset, UK) were used at 10 μM, and dexamethasone (Sigma-Aldrich, Dorset, UK) at 1 μM. Vehicle control (DMSO) was used in all experiments.

### Extraction of total RNA and mRNA quantification

Cells were harvested into TRIzol reagent (Invitrogen,UK) and total RNA was isolated using standard protocols. RNA (1 μg) was reverse-transcribed using Precision reverse transcription kit (Primer Design, Southampton, UK) according to the manufacturer's instructions. cDNA was amplified by PCR using Perfect Probe or Double Dye assays (Primer Design, Southampton, UK). Expression levels of cytokines were calculated relative to housekeeping genes GAPDH and UBC using the ΔΔCT method. Viral RNA was quantified using a reference standard (Primer Design, Southampton, UK).

### Cytokine analysis

Protein secretion of IL-8, Interferon gamma-induced protein (IP-10), and Granulocyte macrophage colony-stimulating factor (GM-CSF) into cell-conditioned media was measured by ELISA (R&D, Abingdon, United Kingdom), following the manufacturer's instructions.

### Statistical analysis

Normality of distribution was assessed using the Shapiro Wilk test (Sigmaplot) and parametric (ANOVA) or non parametric (Friedman repeated measures ANOVA on Ranks) tests undertaken for within group comparisons, as appropriate to detect any overall changes with different treatments. Where significant differences were identified, pairwise tests (paired T-test (T-test) or Wilcoxon signed rank test (Wilcoxon) respectively were used to investigate between-treatment differences. To assess to possibility of synergistic effects, the sum of cytokine release of single treatments (Th2 and virus) and cytokine release observed in combined treatment was compared using the same methodological approaches. P<0.05 was considered statistically significant. Whiskers on boxplots represent 5^th^-95^th^ centile.

## Results

### Th2 cytokines enhance RV16-induced IP-10 release

**Figure 1:**
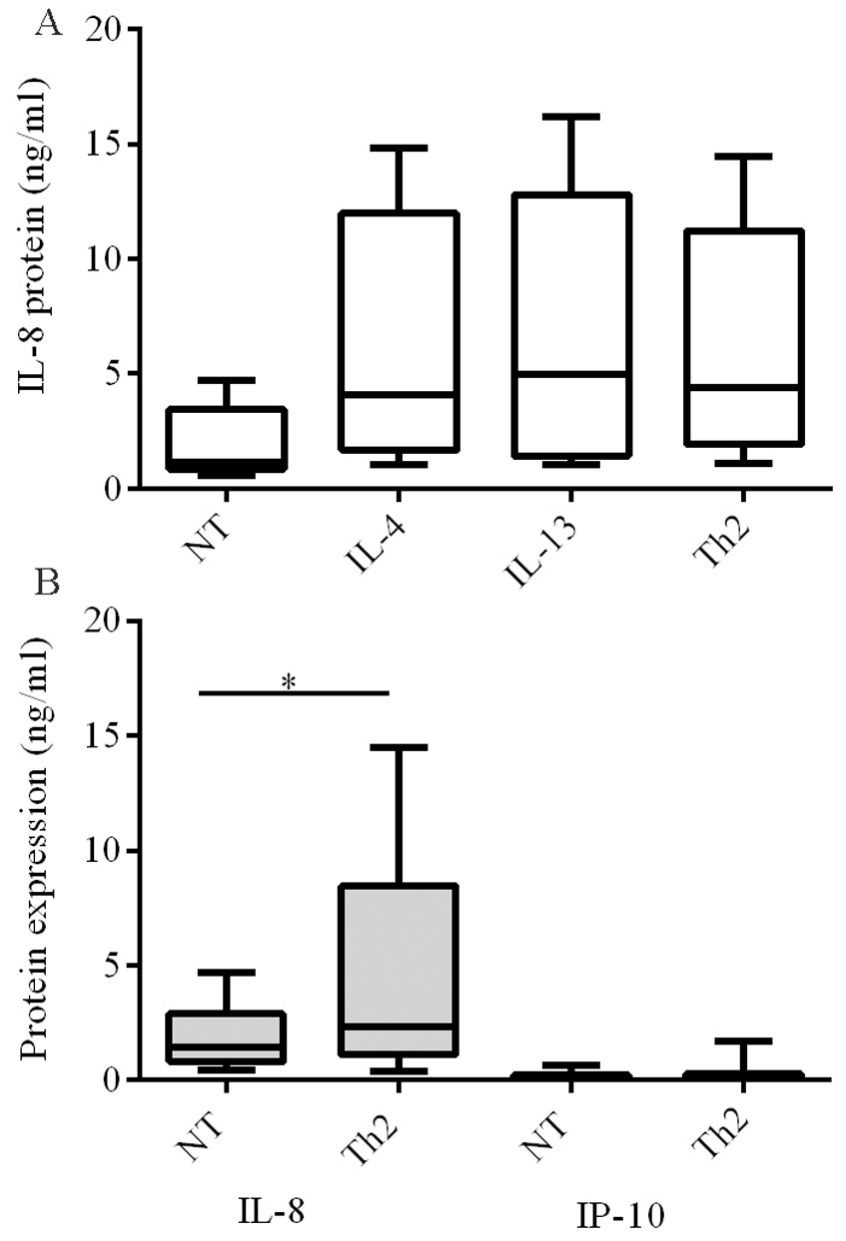
Induction of IL-8 but not IP-10 in response to Th2 stimulation. ppBECs were treated for 24-13 (10 ng/ml) and IL-4 (10 ng/ml) for 24 hours. Protein secretion in cell supernatant was measured by ELISA (R&D). Statistical analysis 1A n = 9 ANOVA P<0.05. 1B n = 15, Wilcoxon, p<0.05. NT-no treatment.

Upregulation of IL-8 expression by IL-13 (10 ng/ml) and IL-4 (10 ng/ml) treatment was confirmed by ELISA. Initial experiments showed cultures treated with IL-4, IL-13 or both cytokines released significantly higher amounts of IL-8 than no treatment (NT) controls (p<0.001, Figure 1A, n = 9) but there was no difference between treatments. This demonstrates that the cells, originating from subjects without asthma, are responsive to Th2 cytokines. The combination of IL-13 and IL-4 induced expression of IL-8 (Figure 1B, p<0.05, n = 15) but not IP-10.

**Figure 2:**
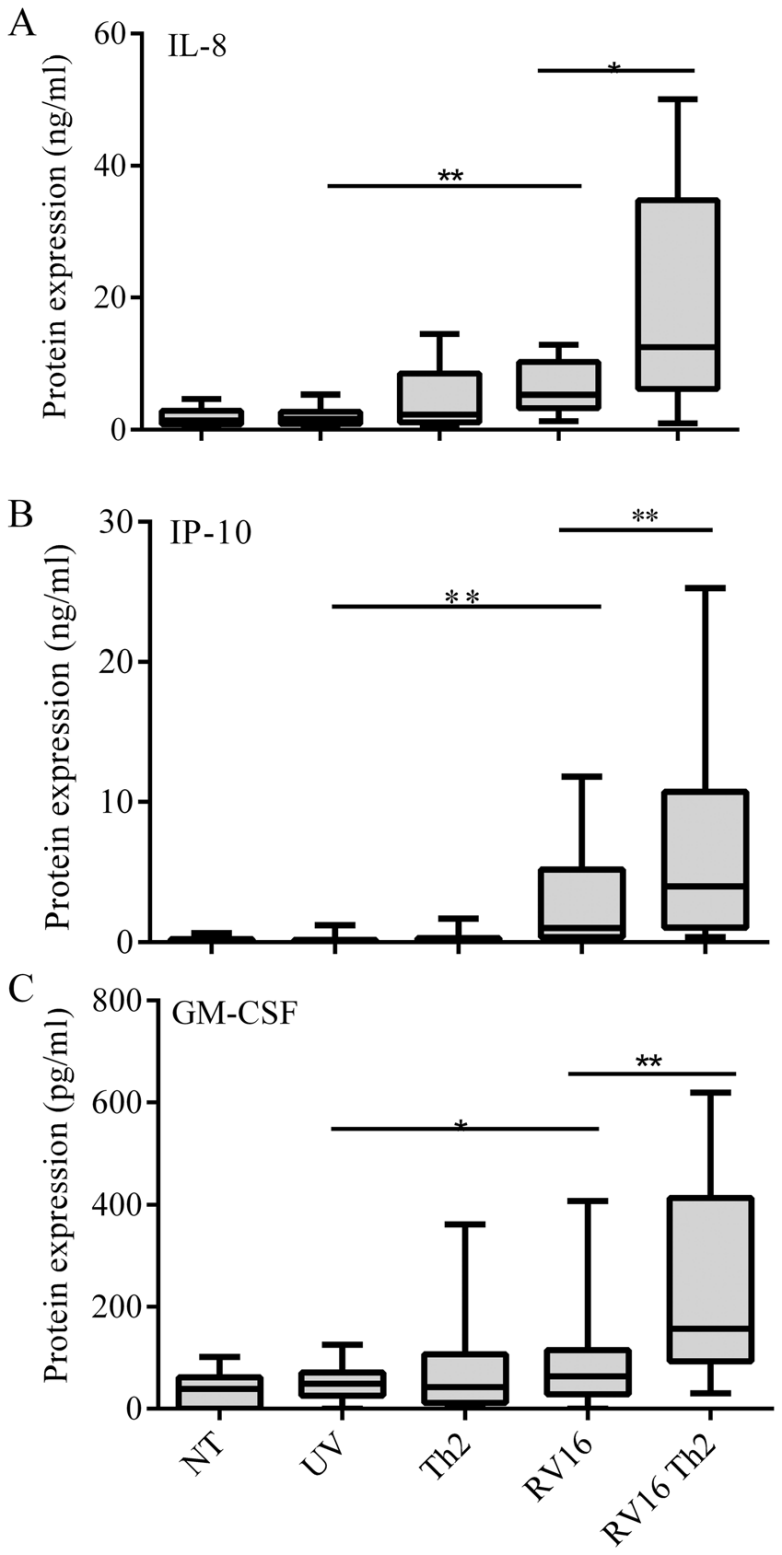
Synergistic increase in virus-induced IP-10 release in the presence of Th2 cytokines. ppBECs were pretreated with IL-13 + IL-4 or not for 24 hours prior to infection with RV16 at 1×10^6^ TCID_50_ units/10^6^ cells (n = 15). Protein secretion in cell culture supernatants was measured after 24 h using ELISA (R&D). Statistical analysis used ANOVA for within group comparison, followed by 2A) IL-8, T-test, 2B) IP-10, Wilcoxon and 2C) GM-CSF, Wilcoxon.*P<0.05 **P<0.01. NT-no treatment.

We next assessed the effect of Th2 cytokines on RV16-induced inflammatory responses. pBECs were treated with Th2 cytokines, or not, for 24 hours before infecting with RV16 (1×10^6^ TCID_50_ units/10^6^ cells (MOI 1). Both IL-8 (p<0.01) and IP-10 p<0.01) release were significantly stimulated by RV16 infection alone (Figure 2A and 2B respectively). No induction of IL-8 or IP-10 was observed with UV irradiated virus (UV), suggesting cytokine induction to be a result of viral replication. The secretion of IL-8 and IP-10 protein was augmented in cultures pretreated with Th2 cytokines (Figure 2A: IL-8, p<0.05, 2B: IP-10, p<0.01). The increase in virus-induced IP-10 expression was synergistic in the presence of Th2 cytokines compared to the sum of the values of single challenges (p<0.01), whilst the increase in IL-8 was additive. We also investigated GM-CSF because of its involvement with dendritic cell recruitment and maturation, linking innate and adaptive immune responses. We saw no significant increase in protein release in response to Th2 cytokines alone but up-regulation of GM-CSF (p<0.05) was observed in response to virus infection. The presence of RV16 and Th2 cytokines resulted in an additive effect on GM-CSF release (p<0.01) compared to virus alone (Figure 2C).

### Viral replication is not moderated by Th2 cytokines

**Figure 3:**
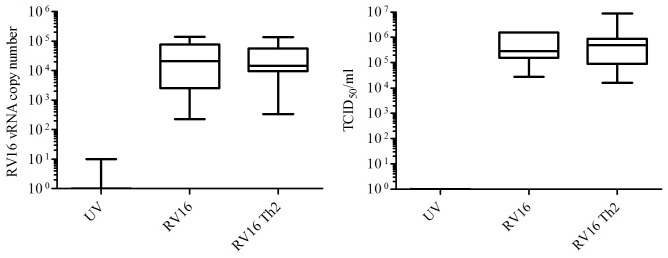
Replication of RV was not modified in the presence of Th2 cytokines. ppBECs were pretreated with IL-13 + IL-4 or not for 24 hours prior to infection with RV16 at 1×10^6^ TCID_50_ units/10^6^ cells. Viral RNA was quantified and expressed as copy number relative to known standards (n = 15). Viral release into the supernatant was measured using TCID_50_ assay (n = 15).

We hypothesised that the presence of Th2 cytokines favoured viral replication, causing the observed increase in IL-8, IP-10 and GM-CSF release. However, we did not see any differences in viral copy number when cells were infected in the absence or presence of the Th2 cytokines (Figure 3) (RV16 copy number (median (range)  =  2.0×10^4^ (2.2×10^2^–1.4×10^5^) *versus* 1.4×10^4^ (3.0×10^2^–1.4×10^5^) n = 15, respectively). Viral titre (virus released into the supernatant) was measured using TCID_50_ and again we could detect no difference between cells pretreated in the absence or presence of Th2 cytokines (RV16 titre (median (range))  =  2.8×10^5^ (2.8×10^4^–1.5×10^6^) *versus* 5×10^5^ (1.5×10^4^–8.9×10^6^) n = 15, respectively). Absence of replicating virus in the UV controls was confirmed using the same methods.

Major group RVs such as RV16 are reported to have less cytotoxicity than minor group virus [20] and in keeping with this we did not see cytopathic effects in our cultures at 24 hours (Figure S1). We measured Lactate Dehydrogenase (LDH), an enzyme found in the cytosol of cells and released into supernatant upon cell damage or lysis. We did not detect a difference in LDH levels between cells infected with virus in the presence of Th2 cytokine compared to virus alone (data not shown).

We also examined expression of ICAM-1, the cellular receptor for RV16. There was no detectable change in expression following 24hr Th2-cytokine treatment. (Figure S2). Together these data are consistent with the view that the observed increases in cytokine expression in the presence of Th2 cytokines is not a consequence of increased entry of virus, or increased viral replication.

### Inhibition of inflammatory pathways

The combined effects of Th2 cytokines and RV16 infection produced different effects depending on the response being measured. IP-10 release was synergistic while IL-8 and GM-CSF release were additive. One explanation could be that these cytokines are stimulated by distinct signaling pathways. We therefore repeated our experiments using inhibitors of PI3K (LY294002) [21] and p38 MAPK (SB203580) pathways [22] and also Dexamethasone [23]. LY294002 is being considered for development as a cancer drug [24] and has potential in respiratory disease. SB203580 was chosen since activation of p38 MAP kinases occurs early in RV infection so participates in signalling cascades controlling cellular responses to cytokines and stress [25]. Dexamethasone was chosen since it is used as an anti inflammatory treatment for asthma and has been shown to inhibit IL-8 in previous studies [26], [27].

**Figure 4:**
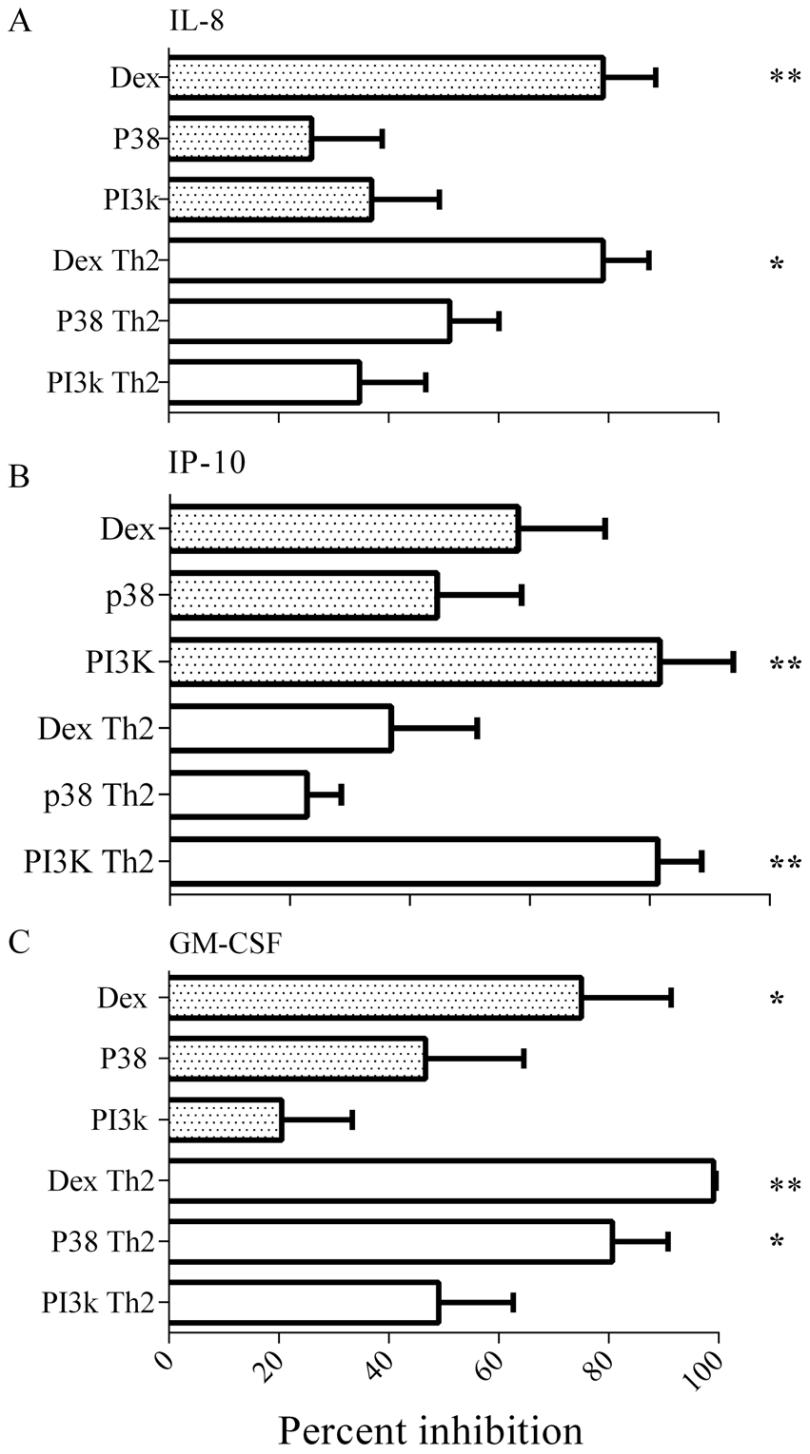
Inhibition of inflammatory pathways suppress virus- induced cytokines. ppBECs were pretreated with IL-13 + IL-4 (clear bars) or not (shaded bars) for 24 hours prior to infection with RV16 (n = 8). Inhibitors were added 30 minutes prior to infection. Protein secretion in cell supernatant was measured using ELISA (R&D). Statistical analysis used ANOVA (*p<0.05) followed by 4A) IL-8, Wilcoxon (absence) or T-test (presence) of Th2 cytokines, 4B IP-10 Wilcoxon (absence) or T-test (presence) of Th2 cytokines and 4C) GM-CSF, T-test. Data were normalized relative to no treatment and cytokine only controls. Total inhibition is shown as 100% and no inhibition as <1%.

A significant change in virus-induced IL-8 and IP-10 protein expression was observed in the presence of inhibitors, with or without Th2 cytokines (ANOVA: IL-8 p = 0.001, IP-10 p = 0.04). Dexamethasone significantly inhibited RV16-induced IL-8 release both in the absence (p = 0.008) or presence (p = 0.03) of Th2 cytokines (Figure 4A). The potent inhibitory effect of dexamethasone on IL-8 release is in keeping with our previous observations [28]. The PI3K inhibitor had only a modest inhibitory effect on IL-8 release but significantly reduced RV16-induced IP-10 expression in the absence (p = 0.016) or presence (p = 0.017) of Th2 cytokines (Figure 4B). Dexamethasone was much less effective at inhibiting RV16-induced IP-10 than the PI3K inhibitor, and the effect was reduced further in the presence of Th2 cytokines. We saw no significant inhibitory effects on IL-8 or IP-10 with the p38 MAPK inhibitor.

RV16-induced GM-CSF expression was almost completely suppressed by dexamethasone both in the absence (p<0.05) or presence (p<0.01) of Th2 cytokines (Figure 4C). Virus replication and release were not altered by the presence of inhibitors in the cultures (Figure S3). This suggests that the inhibitors were acting on signal transduction pathways linked to cytokine expression and was not a result of suppression of virus replication.

We show that RV-induced IP-10 release is synergistically enhanced in the presence of Th2 cytokines, and that this effect can be blocked by inhibition of the PI3K pathway.

## Discussion

In this study, we used primary epithelial cells from a non-asthmatic pediatric population and experimentally promoted a Th2 environment using exogenous Th2 cytokines. We show that, in undifferentiated pediatric bronchial epithelial cell cultures, Th2 cytokines do not increase entry or replication of virus but do augment the release of inflammatory mediators following RV infection.

The complexity of interactions between environment, host and pathogen make it difficult to assess the contribution of individual components of asthma or atopy but several attempts have been made to tease apart these interactions [29]–[31]. For simplicity we chose to use non-differentiated cultures to mimic the basal cells of the pseudostratifed bronchial epithelium [32] with a combination of both cytokines to more closely model *in vivo* conditions [14], [33], [34]. It is known that these cells are exposed in asthmatic bronchial epithelium due to epithelial damage and/or fragility [35]. When we designed the study we did not collect data on atopy since this would incur additional skin and blood tests which we felt would be unreasonable in such a young cohort. We recognize that this is a limitation of the study. Further, to our knowledge there are no data comparing responses of very young children with older teenagers. The wide age range used in this study was unavoidable given the difficulty in recruiting pediatric subjects and we cannot exclude age effects on the responses of the cells.

Our aim was to investigate whether Th2 cytokines, IL-13 and IL-4, modulate the effects of RV infection in bronchial epithelial monolayers. We confirmed previously reported RV-induced up-regulation of inflammatory cytokines IL-8 and IP-10 observed in a cell line [36] and in pBECs taken from adult subjects (Figure 2) [28]. In the study by Wark *et al* rhinovirus induced IP-10 and IL-8 expression of pBECs from adult subjects with allergic asthma was not significantly different to nonatopic healthy controls [28] and this may also be true of pediatric cultures. To our knowledge a similar comparison in a pediatric population has not been performed and unfortunately samples were not readily available for comparison in this study [19], [37].

Using ppBECs from non asthmatic subjects we demonstrate that infection in the presence of Th2 cytokines stimulated a significant increase in IL-8, IP-10 and GM-CSF release. This appeared to be unrelated to a direct effect of the Th2 cytokines on viral replication, since no significant increase in viral RNA expression or viral titre was observed. Most importantly, the effect of RV infection and Th2 cytokines on IP-10 release was synergistic and this effect could be inhibited by blocking the activity of PI3K. Further investigations are important to ascertain the basis of this enhancement, but this is beyond the scope of the present study.

It has been reported that expression of ICAM-1, the cellular receptor for major group RVs such as RV16 is up-regulated by IL-4 and IL-13 following 24 hour incubation [14], [34]. Such an increase in ICAM-1 would be expected to make the cells more vulnerable to RV16 infection, with increased binding and entry of virus into the cells leading to enhanced viral replication. We saw variability in the level of ICAM-1 expression in cultures taken from a small subset of 6 children (ages 1–15) used in the study but we did not see any significant up-regulation of ICAM-1 expression following 24 hr stimulation with Th2 cytokines. Whilst these results differ from some previous publications, this may be explained by the different models used [14], [34].

We did not observe differences in cytopathic effects or LDH release in our cultures at 24 hours (Figure S1) suggesting that the increases in IL-8, IP-10 and GM-CSF were not caused by increased cell death or enhanced viral replication. This supports the findings of Wood *et al* who performed a study in an asthmatic population presenting to hospital with acute asthma exacerbation. They reported that expression of IP-10 is maintained when non-replicating virus is present (ie. viral persistence) and concluded that cellular inflammation caused by viral infection rather than viral replication itself was responsible for the clinical manifestations of a viral induced exacerbation of asthma [38].

We have previously reported that IP-10 expression is much less sensitive to suppression by dexamethasone and that IP-10 levels in serum remain elevated in subjects admitted to the emergency room with a virus-induced asthma exacerbation [28]. We found that dexamethasone was a poor inhibitor of IP-10 release from RV16 infected pBECs but confirmed potent inhibition of IL-8 release, in keeping with other studies [26], [27].

We show that IP-10 release can be blocked by inhibition of PI3K. IP-10 expression is known to be corticosteroid refractory, and our finding that inhibition of PI3K in pBECs potently suppresses IP-10 expression may have therapeutic implications, having the potential to attenuate IP-10 and bring inflammation under control where dexamethasone has no effect. PI3Ks play important roles in tumorigenesis, so there is considerable interest in development of PI3K inhibitors as cancer potential therapeutic agents [39]. LY294002 acts as a competitive ATP binder [40] and has significant *in vivo* antitumor efficacy [24]. Recent studies elucidating the crystal structure of LY294002 provides a basis for development of its therapeutic potential [41].

In conclusion we show that Th2 cytokines, IL-13 and IL-4, synergistically enhance release of RV-induced IP-10, independent of replicating virus. This release can be inhibited by blocking the PI3K pathway.

## Supporting Information

Figure S1 **Phenotypes of cell cultures under the different conditions.** pBECs were pretreated with IL-13 + IL-4 or SFM for 24 hours prior to infection with RV16 at 1×10^6^ TCID_50_ units/10^6^ cells (n = 15). After 1 hr, infection medium was removed and the cells washed with PBS. Starvation medium was replaced +/− cytokines. No differences were seen between the different treatments. Pictures are representative of all the cultures.
(TIF)

Figure S2 **ICAM-1 receptor expression was not altered following 24hr stimulus with Th2 cytokines.** pPBECs were treated for 24 hours with IL-13 10 ng/ml, IL-4 10 ng/ml or both. ICAM-1 expression was assessed using flow cytometry. Data are plotted as ICAM-1 expression relative to isotype control, or as percent positive cells n = 6. No treatment controls were included for comparison.
(TIF)

Figure S3 **Replication of virus was not moderated by inhibitors.** pBECs were pretreated with IL-13 + IL-4 or SFM for 24 hours prior to infection with RV16 at 1×10^6^ TCID_50_ units/10^6^ cells (n = 8). Inhibitors were added 30 minutes before infection. After 1 hr, infection medium was removed and the cells washed with PBS. Starvation medium was replaced +/− cytokines and/or inhibitors. Viral release into cell culture supernatants was measured using TCID_50_ assay. Expression of viral RNA was quantified using RT-qPCR and expressed as copy number relative to known standards.
(TIF)
