# Supplementary figures and images for: Rhinovirus-16 Induced Release of IP-10 and IL-8 Is Augmented by Th2 Cytokines in a Pediatric Bronchial Epithelial Cell Model
Source: PLoS One. 2014 Apr 4;9(4):e94010. doi: 10.1371/journal.pone.0094010 (PMC3976391; doi:10.1371/journal.pone.0094010)

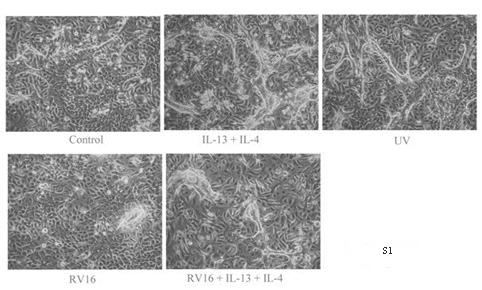

Supplement: Figure S1 — Phenotypes of cell cultures under the different conditions. pBECs were pretreated with IL-13 + IL-4 or SFM for 24 hours prior to infection with RV16 at 1×10^6^ TCID50 units/106 cells (n = 15). After 1 hr, infection medium was removed and the cells washed with PBS. Starvation medium was replaced +/− cytokines. No differences were seen between the different treatments. Pictures are representative of all the cultures. (TIF) [file pone.0094010.s001.tif]

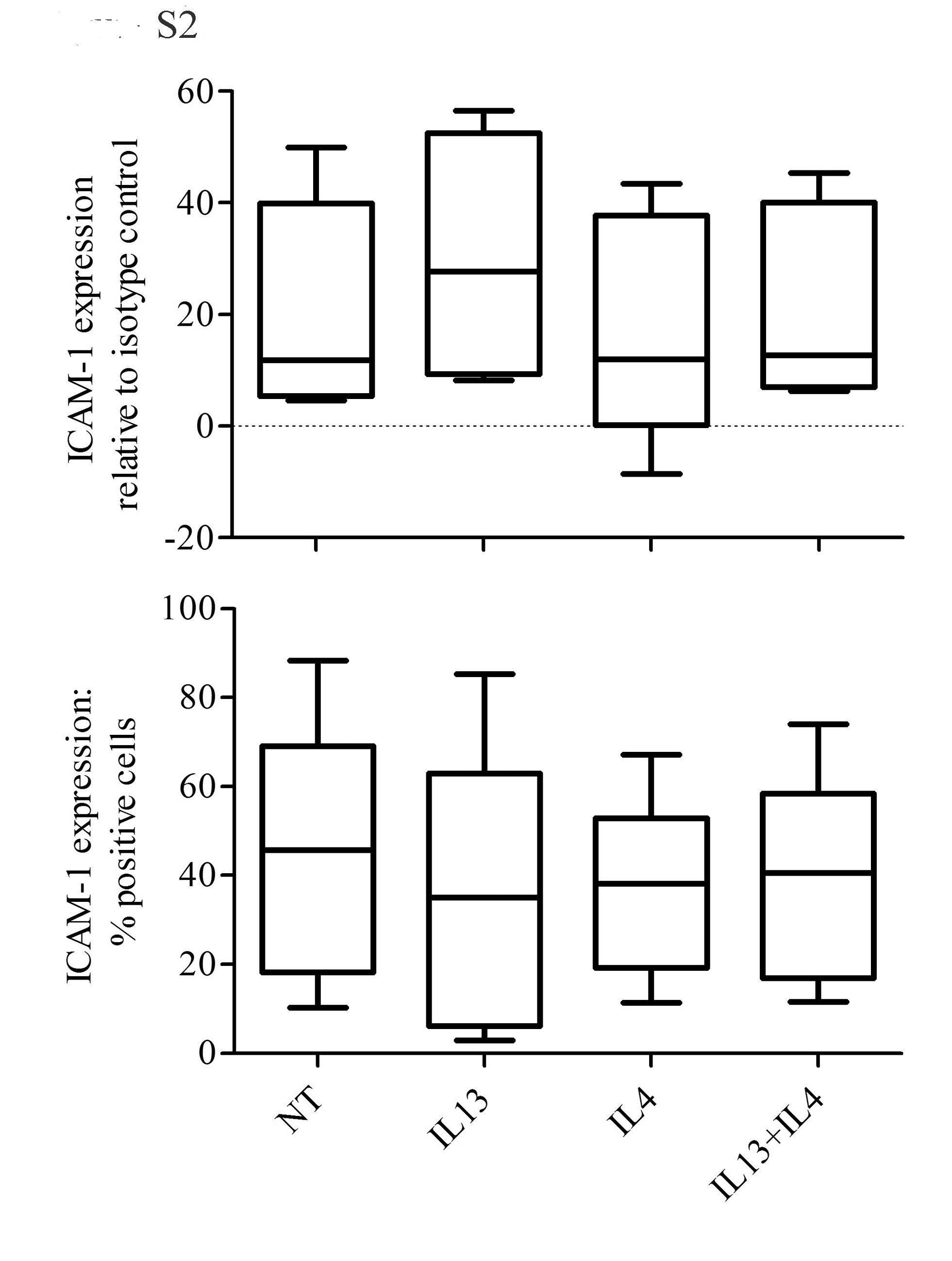

Supplement: Figure S2 — ICAM-1 receptor expression was not altered following 24hr stimulus with Th2 cytokines. pPBECs were treated for 24 hours with IL-13 10 ng/ml, IL-4 10 ng/ml or both. ICAM-1 expression was assessed using flow cytometry. Data are plotted as ICAM-1 expression relative to isotype control, or as percent positive cells n = 6. No treatment controls were included for comparison. (TIF) [file pone.0094010.s002.tif]

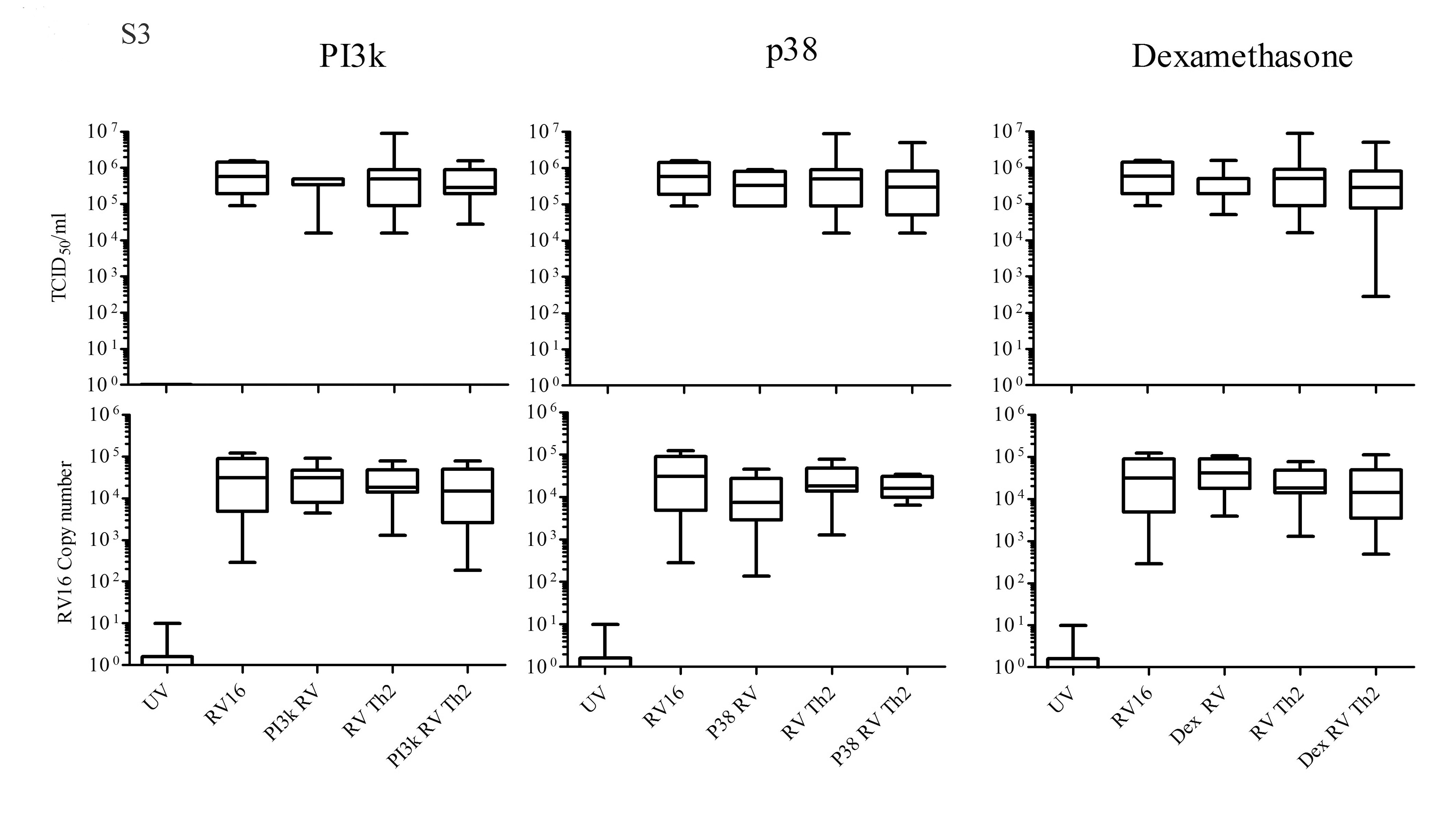

Supplement: Figure S3 — Replication of virus was not moderated by inhibitors. pBECs were pretreated with IL-13 + IL-4 or SFM for 24 hours prior to infection with RV16 at 1×10^6^ TCID50 units/106 cells (n = 8). Inhibitors were added 30 minutes before infection. After 1 hr, infection medium was removed and the cells washed with PBS. Starvation medium was replaced +/− cytokines and/or inhibitors. Viral release into cell culture supernatants was measured using TCID50 assay. Expression of viral RNA was quantified using RT-qPCR and expressed as copy number relative to known standards. (TIF) [file pone.0094010.s003.tif]
